# Within-person modeling of postprandial glucose using multimodal wearable data

**DOI:** 10.3389/fdgth.2026.1847884

**Published:** 2026-06-12

**Authors:** Zilu Liang

**Affiliations:** 1 Ubiquitous and Personal Computing Laboratory, Kyoto University of Advanced Science (KUAS), Kyoto, Japan; 2 Institute of Industrial Science (IIS), The University of Tokyo, Tokyo, Japan

**Keywords:** CGM, continuous glucose monitoring, macro-nutrients, mixed-effects modeling, multimodal dataset, postprandial glucose, within-person analysis

## Abstract

The widespread adoption of continuous glucose monitoring (CGM) and wearable sensing technologies has enabled large-scale collection of high-resolution physiological and behavioral data in real-world settings. However, the analytical frameworks needed to translate these data into actionable, individualized insights remain limited. In particular, many existing approaches rely on population-level analysis or controlled experimental designs, which often fail to capture intra-individual variability in everyday life. To address this sensing–analysis gap, this study investigates within-person, meal-level predictors of postprandial glucose dynamics using multimodal data collected under free-living conditions. We analyzed outcomes including peak glucose, time to peak, and area under the glucose curve above 140 mg/dL. Predictors encompassed macronutrient composition, baseline glucose, meal timing, and short-term wrist movement variability derived from wearable sensors. Linear mixed-effects models were constructed with continuous predictors centered within individuals to explicitly capture within-person effects. Net carbohydrate intake showed the strongest association with postprandial glucose magnitude (p<0.001), whereas total fat intake was associated with delayed glucose responses (p=0.024), suggesting that glucose magnitude and response timing may reflect distinct physiological patterns. Baseline glucose was consistently associated with all outcomes (p<0.001). In addition, greater short-term wrist movement variability was linked to reduced glucose excursions, suggesting the potential relevance of behavioral context in postprandial glucose variability. These preliminary findings demonstrate the potential utility of integrating multimodal wearable and behavioral data to characterize individualized postprandial glucose responses in real-world settings.

## 1 Introduction

The rapid proliferation of wearable sensing technologies, including continuous glucose monitors (CGM) and wrist-worn devices, has fundamentally transformed data collection in digital health [1–3]. These systems enable continuous, multimodal monitoring of physiological and behavioral signals under free-living conditions, and are generating high-resolution longitudinal datasets at an unprecedented scale [4–6]. However, the development of analytical frameworks capable of translating these data into actionable, individualized insights has lagged behind. Most existing studies continue to rely on controlled experimental designs or population-level analysis, which do not fully leverage the richness of within-person variability. As a result, a sensing–analysis gap has emerged, which limits the ability of digital health systems to deliver personalized, context-aware interventions. While wearable technologies have made continuous sensing widely accessible, the ability to extract meaningful, personalized insights remains limited. In this context, the central challenge in digital health is not just data acquisition, but more importantly, data analysis and interpretation. Addressing this imbalance is essential for unlocking the full potential of wearable-based health monitoring systems.

One important application of CGM is the characterization of postprandial glucose dynamics [7–9], which are central to diabetes prevention and management and increasingly recognized as clinically relevant even among individuals without diagnosed diabetes [10, 11]. Elevated postprandial glucose excursions are associated with impaired glucose tolerance and may represent an early marker of metabolic dysregulation preceding overt diabetes [12, 13]. Understanding how nutritional composition relates to both the magnitude and timing of postprandial glucose responses is therefore essential for informing dietary recommendations and enabling adaptive digital health technologies.

Despite this importance, much of the existing literature on postprandial glucose dynamics is derived from controlled laboratory studies using standardized meals or predefined macronutrient manipulation [13, 14]. While these approaches offer strong internal validity, they do not capture the variability of real-world eating behavior, where meals differ widely in composition, portion size, timing, and surrounding behavioral context. Moreover, many studies rely on cross-sectional or between-person analysis, which limits their ability to inform individualized glycemic regulation [14–16]. Consequently, it remains unclear how meal-to-meal variations within the same individual influence postprandial glucose in everyday life.

Recent advances in CGM and wearable sensing technologies provide high-resolution data that create new opportunities to study glycemic responses at the level at which interventions are ultimately applied. However, realizing this potential requires analytical approaches that explicitly model intra-individual variability and contextual factors.

This study addresses this gap by leveraging multimodal, free-living CGM and wearable sensing data to examine within-person, meal-level predictors of postprandial glucose dynamics. Specifically, we investigate how deviations in meal composition and contextual factors from the typical patterns of an individual are associated with variations in peak glucose magnitude and time to peak. Rather than emphasizing between-person differences, the present study focuses on within-person variability and how changes relative to an individual’s own baseline correspond to variations in glycemic responses.

The primary contribution of this work is to explore the potential value of ecologically valid, within-person modeling using multimodal wearable data for characterizing postprandial glucose responses in everyday settings. By leveraging repeated, meal-level observations within individuals, we observed that different aspects of glycemic response magnitude and timing were associated with partially distinct behavioral and nutritional factors. These preliminary findings suggest that separating response magnitude from temporal dynamics may provide a useful analytical perspective for understanding individual metabolic variability under free-living conditions.

More broadly, this study contributes to the ongoing shift in digital health analytics from population-level inference toward individualized, within-person modeling. While the present findings should not be interpreted as conclusive, they highlight the potential utility of modeling intra-individual variability using multimodal wearable data to support future personalized and context-aware digital health applications.

## 2 Method

### 2.1 Dataset and preprocessing

This study utilized the BIG IDEAs dataset [17, 18], which integrates CGM, wearable physiological sensing, and self-reported dietary intake collected under free-living conditions over 8–10 days via dietary logging. Interstitial glucose was measured using the Dexcom G6 system, while physiological signals including tri-axis acceleration (ACC), electrodermal activity (EDA), blood volume pulse (BVP), and skin temperature (TEMP) were recorded using an Empatica E4 wristband. Dietary intake was captured via time-stamped food logs. The Dexcom G6 system includes a 2-h initialization period following sensor insertion during which glucose readings are unavailable; therefore, data collection and analysis began after completion of this warm-up period. Raw physiological and glucose signals were processed using vendor-level filtering procedures provided in the original dataset pipeline. Additional details regarding the study protocol, sensor configuration, and preprocessing procedures are described in the original dataset publications [17, 18].

Food log entries corresponding to the same meal were merged based on timestamps, and macronutrient values were aggregated to derive meal-level nutritional features. For each meal, CGM and wearable signals were aligned to the reported meal onset time and truncated to a 2-h postprandial window, which is consistent with established physiological response periods in prior CGM studies [12, 14–16].

Postprandial glucose outcomes were derived from CGM data, including peak glucose (mg/dL), time to peak glucose (min), and hyperglycemic exposure quantified as the area under the glucose curve above 140 mg/dL (AUC${}_{140}$), a clinically relevant threshold for postprandial hyperglycemia [16]. Predictor variables encompassed meal composition, prior-meal context, and short-term physiological signals. EDA and BVP were excluded from subsequent analysis due to negligible associations with glucose outcomes in our pilot analysis.

The final set of meal-level variables included:

- Postprandial outcomes: peak glucose (mg/dL), time to peak (min), and AUC${}_{140}$ (mg/dL⋅min).
- Nutritional variables: net carbohydrate, dietary fiber, total fat, and protein intake (g).
- Contextual and physiological variables: time of day (morning: 06:00–11:00; afternoon: 11:00–18:00; evening: 18:00–06:00), time since previous meal (min), baseline glucose (mean CGM value in the 5 min prior to meal onset, mg/dL), and short-term wrist activity variability (standard deviation of the root mean square of tri-axis ACC in the 30 min following meal onset, m/s${}^{2}$).

### 2.2 Statistical analysis

To model meal-level variability while accounting for repeated observations, we employed linear mixed-effects regression models with participant-specific random intercepts. This approach separates within-person dynamics from between-person differences, which aligns with the goal of personalized modeling in digital health contexts.

All continuous predictors were centered within individuals so that values represent deviations from the typical level of each participant. As shown in Equation 1, this within-person centering isolates meal-specific effects and reduces confounding by time-invariant individual characteristics. Formally, for subject i, meal j, and predictor k:

$$x_{i,j,k}^{\left(W\right)}=x_{i,j,k}-{\bar{x}}_{i,k},$$ (1)

where ${\bar{x}}_{i,k}$ denotes the subject-specific mean of predictor k. The centered predictors were subsequently standardized using z-score scaling to facilitate coefficient comparison across predictors, improve numerical stability, and reduce multicollinearity in interaction analysis.

For each outcome variable, we fit the following model shown in Equation 2:

$$y_{i,j}=\beta_{0}+\sum_{k=1}^{K}\beta_{k}x_{i,j,k}^{\left(W\right)}+u_{i}+\varepsilon_{i,j},$$ (2)

where $y_{i,j}$ is the postprandial outcome for subject i at meal j, $\beta_{k}$ are fixed-effect coefficients representing within-person associations, $u_{i}$ is a subject-specific random intercept, and $\varepsilon_{i,j}$ is the residual error.

The random intercept $u_{i}$ captures between-subject differences in baseline outcome levels and was assumed to follow a normal distribution with mean zero and variance $\sigma_{u}^{2}$. Residual errors $\varepsilon_{i,j}$ were assumed to be independently and normally distributed with mean zero and variance $\sigma^{2}$. All models were estimated using maximum likelihood and included time of day as a categorical covariate to account for potential diurnal variation in glucose dynamics.

The primary analysis modeled all predictors as fixed effects, representing average within-person associations across participants. Because the study was designed to characterize intra-individual meal-related dynamics rather than between-subject population effects, the analysis focused primarily on within-person variability.

As an exploratory extension, additional analysis was conducted to examine the robustness and potential heterogeneity of the observed associations. First, a random-slope model was estimated for peak glucose by allowing the within-person effect of net carbohydrate intake to vary across participants while retaining fixed slopes for the remaining predictors. Net carbohydrate intake was selected because it represented the primary nutritional variable of interest and demonstrated the strongest associations in the primary models. Random slopes were not estimated for all predictors because doing so would substantially increase model complexity relative to the limited number of participants and could lead to overparameterization and unstable covariance estimation. Second, exploratory interaction analysis was performed by including interaction terms between within-person net carbohydrate intake and concurrent dietary fiber, fat, and protein intake to evaluate potential non-additive associations among meal macronutrients. Finally, leave-one-participant-out (LOPO) sensitivity analysis was conducted by repeatedly refitting the mixed-effects models after excluding one participant at a time to assess the robustness of the observed associations. Given the exploratory nature of these secondary analyses and the limited sample size, only a brief summary of the findings is presented in Section 3.2, with detailed results provided in the Supplementary Material. All analysis were conducted in Python 3.8.8 using the statsmodels library.

## 3 Results

### 3.1 Descriptive statistics

**Figure 1:**
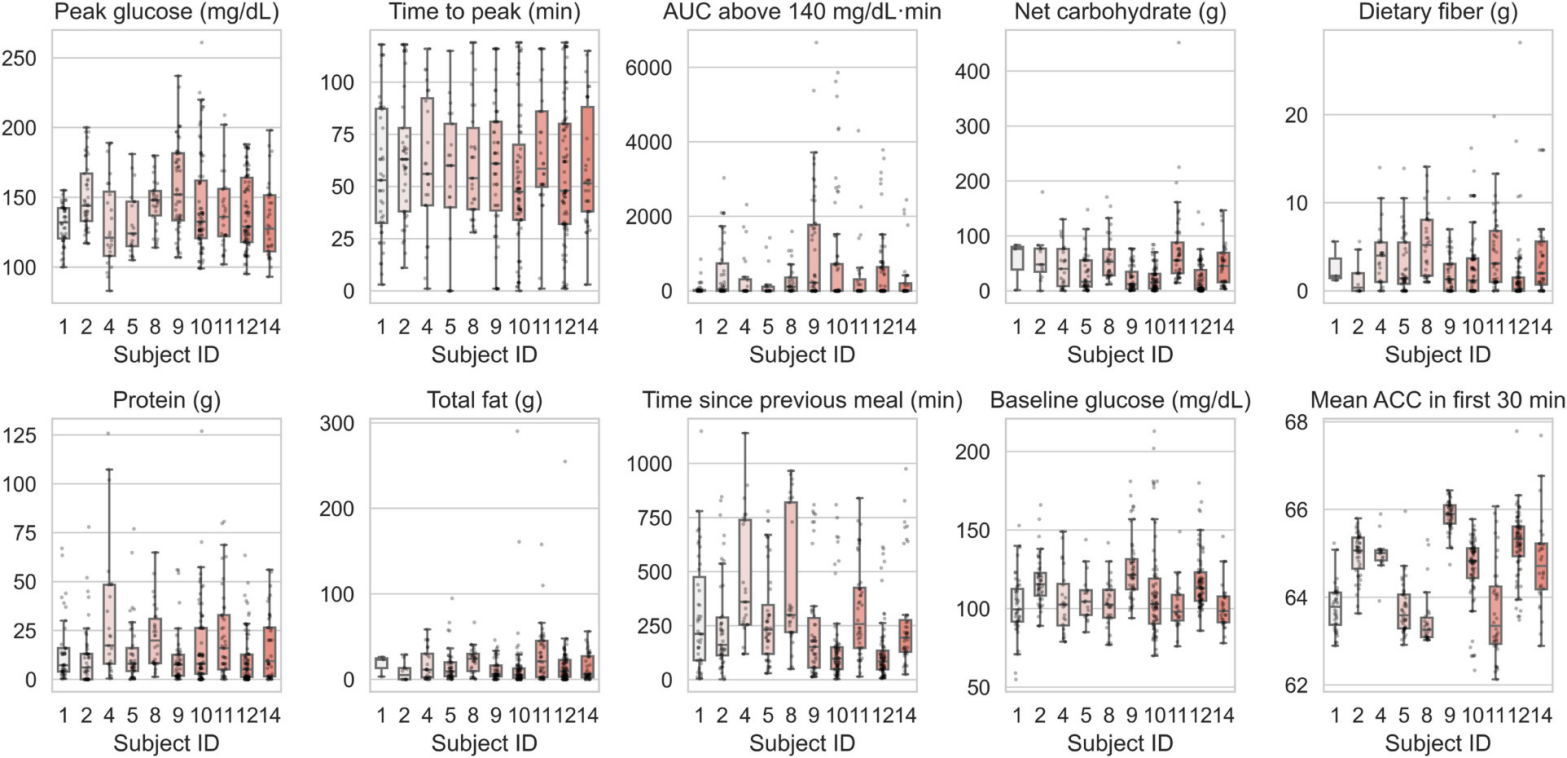
Distributions of postprandial glucose outcomes and meal-level nutritional and contextual predictors across participants.

The final sample included 485 meals from 10 participants (5 male, 5 female; age 35–65 years; HbA1C 5.2%–6.4%). Participants contributed a median of 45 meals (range: 25–82). Figure 1 illustrates the distributions of postprandial glucose outcomes and meal-level predictors across participants under free-living conditions, while detailed descriptive statistics are provided in the Supplementary Material. Substantial within-person variability was observed across all variables. After excluding observations with missing features, 267 meals were retained for regression analysis.

Postprandial glucose responses exhibited considerable dispersion. Peak glucose values typically ranged from approximately 100 to 180 mg/dL, with occasional excursions exceeding 200 mg/dL. Time to peak glucose varied between approximately 30 and 90 min, with pronounced within-individual variability, indicating that both the magnitude and timing of glycemic responses fluctuate meaningfully across meals.

Nutritional intake also showed wide within-person variation. Net carbohydrate intake spanned from near 0 to over 100 g per meal, with occasional higher values, while protein and fat intake displayed similarly broad distributions. Dietary fiber intake was generally lower in absolute magnitude but remained variable relative to individual baselines.

Contextual factors further contributed to variability in the dataset. Time since the previous meal exhibited substantial dispersion, and baseline glucose typically ranged from approximately 80 to 130 mg/dL with moderate intra- and inter-individual variation. In contrast, short-term wrist activity following meal onset showed comparatively lower variability.

### 3.2 Postprandial glucose responses and within-subject predictors

Linear mixed-effects models identified distinct within-person predictors of postprandial glucose magnitude and timing under free-living conditions (Table 1). All models adjusted for time of day to account for diurnal variation. Given the modest number of participants, the findings are interpreted as within-person associations reflecting meal-to-meal variability. Multicollinearity was low across predictors (VIF range: 1.05–1.79).

**Table 1:** Within-person associations between meal-level predictors and postprandial glucose outcomes.

Predictor (within-person)	Peak glucose (mg/dL)	Time to peak (min)	AUC${}_{140}$ (mg/dL⋅min)
Net carbohydrates	12.77 (1.85)${}^{***}$	2.97 (2.44)	453.54 (79.97)${}^{***}$
Dietary fiber	−2.59 (1.54)${}^{†}$	0.45 (2.02)	−150.20 (66.73)${}^{*}$
Protein	1.07 (1.83)	−1.38 (2.44)	−1.16 (79.01)
Total fat	−0.83 (1.50)	4.47 (1.97)${}^{*}$	−16.70 (64.87)
Time from previous meal (min)	3.27 (1.55)${}^{*}$	3.80 (2.07)${}^{†}$	141.67 (67.10)${}^{*}$
Baseline glucose (5 min)	13.69 (1.26)${}^{***}$	−9.58 (1.65)${}^{***}$	320.43 (54.51)${}^{***}$
Wrist movement variability (30 min SD)	−5.43 (1.38)${}^{***}$	0.11 (1.81)	−130.37 (59.75)${}^{*}$

Values are fixed-effect coefficients β (standard error) from linear mixed-effects models with participant-specific random intercepts. All predictors were centered within participant, such that coefficients represent within-person associations. Models were adjusted for time of day. ${}^{***}p<0.001$, ${}^{**}p<0.01$, ${}^{*}p<0.05$, ${}^{†}p<0.10$.

Net carbohydrate intake emerged as the strongest predictor of postprandial glucose magnitude. Meals containing more carbohydrates than an individual’s typical intake were associated with higher peak glucose (β=12.77, p<0.001) and substantially greater hyperglycemic exposure (β=453.54, p<0.001), but were not associated with time to peak glucose. Dietary fiber intake showed a modest attenuating effect on glucose magnitude, with higher-than-usual intake associated with reduced AUC${}_{140}$ (β=−150.20, p=0.024) and a marginal reduction in peak glucose (β=−2.59, p=0.093), but no effect on timing. In contrast, total fat intake was associated with delayed glucose responses. Higher-than-usual fat intake was linked to longer time to peak glucose (β=4.47, p=0.024) but was not associated with glucose magnitude.

Contextual factors also contributed to variability. Baseline glucose was among the most consistent predictors across outcomes. Higher baseline glucose relative to an individual’s usual level was associated with higher peak glucose (β=13.69, p<0.001), greater hyperglycemic exposure (β=320.43, p<0.001), and shorter time to peak (β=−9.58, p<0.001). Longer-than-usual intervals since the previous meal were associated with slightly higher peak glucose (β=3.27, p=0.035) and AUC${}_{140}$ (β=141.67, p=0.035), with a trend toward delayed peak timing. Short-term wrist activity variability following meal onset was associated with lower peak glucose (β=−5.43, p<0.001) and reduced AUC${}_{140}$ (β=−130.37, p=0.029), but not with time to peak.

ICC analysis based on null random-intercept models indicated small but non-negligible participant-level clustering for peak glucose (ICC = 0.08) and AUC${}_{140}$ (ICC = 0.05), supporting the use of mixed-effects models to account for repeated observations nested within participants. On the other hand, time-to-peak glucose exhibited negligible between-subject variance, suggesting that variability in this outcome was primarily driven by meal-level fluctuations rather than stable individual differences. Nevertheless, mixed-effects models were retained because repeated measurements violate the independence assumption of ordinary regression models.

Furthermore, exploratory random-slope analysis on peak glucose, which allows the within-person effect of net carbohydrate intake to vary across participants, demonstrated substantially improved model fit compared with the random-intercept model (ΔAIC=−26.1; ΔBIC=−18.7), suggesting potential heterogeneity in carbohydrate-related glucose responses across individuals (see Supplementary Material). Due to the limited sample size, however, random slopes were evaluated only for net carbohydrate intake, while more complex random-slope structures were left out for future studies with larger cohorts.

In contrast, inclusion of interaction terms did not substantially improve model fit, with the simpler additive models generally demonstrating lower AIC and BIC values across outcomes (see Supplementary Material). Most interaction terms were non-significant, suggesting that additive associations accounted for the majority of the observed variability in this dataset.

Finally, LOPO sensitivity analysis indicated that the primary associations remained highly stable across all iterations. Specifically, the within-person association between net carbohydrate intake and peak glucose remained consistently positive (β range: 12.55 to 16.30), while baseline glucose remained strongly positively associated with peak glucose (β range: 13.39 to 15.54). Wrist activity variability also consistently showed negative associations with peak glucose (β range: −5.85 to −3.93). Although the statistical significance of dietary fiber varied modestly across iterations, the estimated direction of association remained consistently negative. These findings suggest that the observed associations were not driven solely by any single participant in the present sample.

## 4 Discussion

### 4.1 Differential determinants of glucose magnitude and timing

Clinically, postprandial peak capillary plasma glucose values are generally recommended to remain below 180 mg/dL [19]. Although participants in the BIG IDEAs study did not have diagnosed diabetes, occasional peaks exceeding 200 mg/dL were observed, suggesting transient impairments in glucose regulation that may not be captured by fasting or long-term glycemic markers alone. Time to peak glucose ranged from approximately 30 to 90 min, consistent with prior CGM-based observations in mixed-meal contexts [9, 10, 15, 16, 20, 21]. Some participants consumed less than recommended amounts of dietary fiber and protein [22, 23].

This study shows that postprandial glucose magnitude and timing are associated with partially distinct within-person factors under free-living conditions. Overall, nutritional and contextual variables exhibited stronger and more consistent associations with glucose magnitude than with response timing, suggesting that these two dimensions may capture different aspects of postprandial physiology.

Net carbohydrate intake was the strongest factor associated with glucose magnitude. Within individuals, meals containing more carbohydrates than usual were associated with higher peak glucose and greater hyperglycemic exposure. This finding is consistent with prior evidence linking carbohydrate load with postprandial glycemia [14, 15, 20, 21, 24], while extending these observations to within-person, meal-to-meal variability. In contrast, carbohydrate intake was not associated with time to peak glucose, indicating that its association is more pronounced for glucose amplitude than for response timing.

In comparison, fat intake was primarily associated with the temporal dimension of glucose response. Meals containing more fat than usual were associated with a longer time to peak glucose, while no significant association was observed with glucose magnitude. Although some cross-sectional studies have suggested flatter postprandial glucose curves among individuals with higher habitual fat intake [16, 25–27], the present within-person findings suggest that fat-related effects may be more evident in timing than in glucose magnitude.

Dietary fiber intake showed a modest but directionally consistent association with attenuated glucose magnitude. Higher-than-usual fiber intake was associated with lower hyperglycemic exposure above 140 mg/dL and showed a marginal association with lower peak glucose, while no association was observed with time to peak. Although prior studies have reported similar associations between fiber intake and reduced glycemic responses [16, 28, 29], the relatively smaller effect sizes observed here suggest that, in free-living mixed meals, fiber may be a weaker predictor than carbohydrate quantity.

Protein intake was not associated with any postprandial glucose outcome.

These findings indicate that glucose magnitude and timing are associated with distinct patterns of nutritional factors. Rather than serving as interchangeable indicators, these dimensions may provide complementary perspectives on postprandial glucose dynamics in real-world settings.

### 4.2 Associations to baseline state and behavioral context

Beyond macronutrient composition, baseline glycemic state and recent behavioral context were also consistently associated with postprandial glucose responses. Baseline glucose measured in the 5-min pre-meal window emerged as one of the strongest factors across all outcomes. Within individuals, higher baseline glucose was associated with higher peak glucose, greater hyperglycemic exposure, and a shorter time to peak, even after accounting for meal composition. These findings suggest that short-term fluctuations in glycemic state are closely associated with subsequent postprandial responses.

This observation is consistent with prior CGM-based studies reporting associations between preprandial glucose and postprandial excursions, where baseline glucose has been interpreted as reflecting residual effects of prior meals or circadian variation in insulin sensitivity [7, 16]. Associations between fasting glucose and postprandial AUC have also been reported [7, 30]. Extending this literature, the present within-person findings indicate that short-term deviations in baseline glucose are associated with both the magnitude and timing of postprandial responses, independent of meal composition.

In contrast, meal timing relative to the previous meal showed weaker and less consistent associations. Longer-than-usual inter-meal intervals were associated with greater postprandial glucose magnitude, whereas associations with time to peak did not reach conventional levels of statistical significance.

Short-term wrist movement variability during the 30-min window following meal onset was associated with lower peak glucose and reduced hyperglycemic exposure, with no significant association observed for response timing. This metric was intended to capture short-term variability or irregularity in wrist motion during eating episodes rather than absolute movement intensity, structured physical activity, or energy expenditure. The inclusion of this meal-proximal feature was motivated by the aim of characterizing contextual dynamics specific to individual eating episodes, which may reflect behaviors such as meal pacing or gestural activity during food consumption. Accordingly, the observed associations should be interpreted as exploratory and contextual rather than as evidence of a specific physiological mechanism.

### 4.3 Implications for wearable-based digital health systems

These findings have direct implications for the design of wearable-based digital health systems. Although CGM and wearable devices enable continuous monitoring of physiological and behavioral signals, current analytical approaches often fall short in translating these data into individualized and contextual-aware insights [31–33]. In particular, many existing studies are cross-sectional or focus primarily on CGM-derived metrics without incorporating contextual factors [13–16, 34–36].

The observed within-person associations indicate that carbohydrate intake was more strongly associated with glucose magnitude outcomes, whereas fat intake showed stronger associations with response timing. These findings suggest that glucose magnitude and temporal response characteristics may represent related but distinct dimensions of postprandial dynamics. Accordingly, relying exclusively on aggregate CGM metrics, such as peak glucose or time-in-range [35], may overlook potentially relevant variability in response patterns across meals and individuals.

In addition, pre-meal contextual measures showed consistent associations with postprandial outcomes. Baseline glucose levels measured shortly before meals were among the strongest within-person predictors across several models. Although the observational design does not permit causal interpretation, these results suggest that contextual signals available from wearable devices may provide complementary information beyond meal composition alone.

More broadly, the findings support the potential value of context-aware analytical frameworks for wearable-based glucose monitoring. Integrating CGM-derived signals with meal composition, inter-meal timing, and short-window wearable features may help characterize individualized patterns of postprandial glucose variability more effectively than approaches relying on isolated metrics or population-level averages. Future work using larger cohorts, longitudinal intervention designs, and prospective prediction frameworks will be necessary to determine whether these associations can be translated into actionable personalized decision-support systems.

### 4.4 Limitations

Several limitations should be acknowledged when interpreting the findings. First, this study should be considered exploratory and hypothesis-generating rather than broadly generalizable. Although the sample size was sufficient for exploratory within-person analysis, it limits the ability to draw population-level conclusions or reliably estimate between-person effects. Accordingly, the findings should be interpreted as preliminary evidence of within-person associations that require replication in larger and more demographically diverse cohorts. Second, several measurement-related limitations may have influenced the observed associations. CGM sensor accuracy may decline toward the end of the wear period, and no additional quality-control filtering was applied beyond standard Dexcom signal flagging. In addition, dietary intake and meal timing were self-reported and may be subject to estimation error. However, without independent dietary validation, it is not possible to fully characterize the direction or magnitude of these potential errors. Third, while wrist-based activity was included as a contextual factor, it should not be interpreted as a direct measure of metabolically meaningful physical activity or energy expenditure. The metric likely captured a mixture of wrist-related behaviors, including gestural activity, postural transitions, and meal-related movements during the immediate post-meal period. Furthermore, several potentially relevant covariates, including sleep, stress, medication, habitual physical activity, and body composition, were unavailable in the dataset and therefore could not be included in the analysis. These unmeasured factors may have contributed to residual confounding and limit the interpretation of the observed associations. Finally, the observational design precludes causal inference, and unmeasured time-varying factors may also have influenced postprandial responses. All reported findings should therefore be interpreted as associative relationships only.

Despite these limitations, the study demonstrates the feasibility of within-person, context-aware modeling of multimodal wearable data under free-living conditions. Future work using larger and more diverse cohorts, prospective prediction frameworks, and longitudinal intervention designs will be necessary to validate these findings and evaluate their potential applicability in personalized digital health systems.

## 5 Conclusion

This study demonstrates that different dimensions of postprandial glucose dynamics are associated with distinct within-person contextual factors under free-living conditions. Carbohydrate intake and baseline glucose levels were more strongly associated with glucose magnitude outcomes, whereas response timing showed stronger associations with baseline glucose and fat intake. These findings highlight the importance of modeling postprandial glucose as a multidimensional phenomenon and support within-person, meal-level approaches for characterizing individualized glucose variability in everyday settings. More broadly, this work underscores the potential value of context-aware analytical frameworks that integrate wearable sensing, meal composition, and behavioral information to better capture individualized patterns of postprandial glucose responses.

## Data Availability

The original contributions presented in the study are included in the article/Supplementary Material, further inquiries can be directed to the corresponding author/s.
