# Supplementary material for: Within-person modeling of postprandial glucose using multimodal wearable data
Source: Front Digit Health. 2026 Jun 12;8:1847884. doi: 10.3389/fdgth.2026.1847884 (PMC13303617; doi:10.3389/fdgth.2026.1847884)
Supplement: Supplementary file 1 [file Datasheet1.pdf]

Supplementary Material

1 DESCRIPTIVE STATISTICS

Tables S1– S10 present subject-level descriptive statistics for the primary glycemic outcomes, dietary variables, and physiological features included in the analyses. For each participant, the tables report the number of observations (N), minimum, first quartile (Q1), median, third quartile (Q3), and maximum values. These summaries provide an overview of the distribution and inter-individual variability of meal-related glycemic responses, nutritional intake, and wearable-derived physiological measures across the study cohort.

2 EXPLORATORY RANDOM-SLOPE ANALYSIS

To further investigate potential inter-individual variability in meal-related glycemic responses, we conducted an exploratory random-slope analysis in addition to the primary random-intercept models. Specifically, the within-person effect of net carbohydrate intake was allowed to vary across participants while retaining subject-specific random intercepts.

Table S1. Peak glucose (mg/dL).

Subject ID	N	Min	Q1	Median	Q3	Max
1	44	100.0	120.5	132.0	142.2	155.0
2	45	117.0	133.0	144.0	167.0	200.0
4	24	83.0	108.0	121.0	154.0	189.0
5	21	105.0	115.0	124.0	147.0	181.0
8	30	114.0	137.0	148.0	154.5	180.0
9	51	107.0	133.5	152.0	181.5	237.0
10	76	99.0	120.8	132.5	162.0	261.0
11	28	102.0	122.0	136.0	156.0	209.0
12	82	95.0	118.0	129.0	164.0	188.0
14	34	93.0	111.2	127.5	151.5	198.0

Table S2. Time to peak (min).

Subject ID	N	Min	Q1	Median	Q3	Max
1	44	3.0	32.5	53.0	87.2	118.0
2	45	11.0	38.0	63.0	78.0	118.0
4	24	1.0	41.0	56.0	92.2	116.0
5	21	0.0	40.0	60.0	80.0	115.0
8	30	28.0	39.0	54.0	78.0	119.0
9	51	1.0	38.5	61.0	81.0	116.0
10	76	0.0	34.0	47.5	70.0	119.0
11	28	1.0	49.8	58.5	86.0	116.0
12	82	1.0	32.0	48.0	80.0	119.0
14	34	3.0	38.0	51.5	88.0	115.0

Table S3. AUC above 140 mg/dL·min.

Subject ID	N	Min	Q1	Median	Q3	Max
1	44	0.0	0.0	0.0	16.2	852.5
2	45	0.0	0.0	40.0	735.0	3032.5
4	24	0.0	0.0	0.0	308.1	2315.0
5	21	0.0	0.0	0.0	110.0	1425.0
8	30	0.0	0.0	110.0	357.5	1600.0
9	51	0.0	0.0	215.0	1772.5	6670.0
10	76	0.0	0.0	0.0	715.0	5862.5
11	28	0.0	0.0	0.0	304.4	4300.0
12	82	0.0	0.0	0.0	635.0	3790.0
14	33	0.0	0.0	0.0	200.0	2445.0

Table S4. Net carbohydrate (g).

Subject ID	N	Min	Q1	Median	Q3	Max
1	3	1.3	38.9	76.4	79.8	83.3
2	12	0.0	34.8	48.0	76.8	180.0
4	25	0.0	8.5	40.0	76.4	130.5
5	45	0.1	7.5	16.4	55.5	148.0
8	30	11.5	28.4	52.7	76.0	170.8
9	51	0.1	3.6	12.0	34.5	77.0
10	76	0.0	3.8	15.5	30.8	84.2
11	45	14.5	32.0	55.5	88.0	452.2
12	82	0.0	2.8	4.7	37.7	143.6
14	41	3.5	15.5	45.3	69.0	146.7

Table S5. Dietary fiber (g).

Subject ID	N	Min	Q1	Median	Q3	Max
1	3	1.2	1.4	1.7	3.6	5.6
2	12	0.0	0.0	0.3	2.0	5.6
4	25	0.0	1.0	4.0	5.5	14.0
5	45	0.0	0.8	1.4	5.5	13.9
8	30	1.0	1.7	5.2	8.1	14.1
9	51	0.0	0.0	1.3	3.0	10.5
10	76	0.0	0.0	1.2	3.7	16.2
11	45	0.0	1.0	3.1	6.8	19.8
12	82	0.0	0.0	0.2	1.5	28.2
14	41	0.0	0.7	2.0	5.6	16.0

Net carbohydrate intake was selected as the random-slope variable because it demonstrated the strongest fixed-effect association with postprandial glucose outcomes in the primary analyses. Allowing participant-specific slopes for carbohydrate intake is also biologically plausible, as prior personalized nutrition research has shown substantial heterogeneity in glycemic responses to carbohydrate consumption. Due to the limited number of participants ($n = 10$), random slopes were not estimated for all predictors because doing so

Table S6. Protein (g).

Subject ID	N	Min	Q1	Median	Q3	Max
1	44	0.2	4.0	7.2	16.0	67.0
2	46	0.0	0.0	6.0	13.0	78.0
4	25	0.4	8.0	17.3	48.3	125.8
5	45	0.0	4.3	8.0	16.0	77.0
8	30	1.3	8.3	19.9	31.0	64.9
9	51	0.0	1.9	7.6	12.6	56.0
10	76	0.0	2.8	8.0	26.2	127.0
11	45	0.0	5.0	16.0	32.9	80.7
12	82	0.0	1.2	5.0	12.7	63.4
14	41	0.0	1.5	9.3	26.5	56.0

Table S7. Total fat (g).

Subject ID	N	Min	Q1	Median	Q3	Max
1	3	3.3	13.2	23.1	24.6	26.0
2	12	0.0	0.0	5.0	13.3	29.0
4	25	0.1	2.5	11.3	30.0	58.5
5	45	0.0	3.7	9.3	20.0	95.0
8	30	0.4	9.8	24.2	29.8	66.6
9	51	0.0	3.5	5.8	16.1	46.0
10	76	0.0	1.7	5.1	12.8	290.5
11	45	0.0	2.6	21.0	45.0	158.0
12	82	0.0	3.0	9.5	22.6	255.0
14	41	0.0	2.5	6.0	27.1	56.0

Table S8. Time since previous meal (min).

Subject ID	N	Min	Q1	Median	Q3	Max
1	43	5.0	90.0	212.0	475.0	1150.0
2	45	2.0	113.0	162.0	288.0	847.0
4	24	120.0	255.0	360.0	738.8	1140.0
5	44	30.0	120.0	232.5	345.0	780.0
8	29	50.0	220.0	300.0	820.0	966.0
9	50	14.0	56.2	152.5	285.0	810.0
10	75	3.0	49.0	98.0	150.5	810.0
11	44	15.0	147.5	257.5	425.0	840.0
12	81	6.0	48.0	85.0	135.0	614.0
14	40	25.0	128.8	195.5	276.2	975.0

would substantially increase model complexity and risk overparameterization, convergence instability, and unreliable covariance estimation.

Compared with the random-intercept model, the random-slope model demonstrated substantially improved model fit, as indicated by lower Akaike Information Criterion (ΔAIC = -26.1) and Bayesian Information Criterion (ΔBIC = -18.7) values (Table S11). The estimated variance of the random slope further suggested meaningful inter-individual variability in carbohydrate-related glycemic sensitivity.

Table S9. Baseline glucose (mg/dL).

Subject ID	N	Min	Q1	Median	Q3	Max
1	44	55.0	91.8	99.5	112.5	153.0
2	44	89.0	108.2	115.5	122.8	166.0
4	24	79.0	89.2	102.5	115.8	149.0
5	18	85.0	96.0	104.5	111.8	144.0
8	30	77.0	94.2	102.5	112.0	142.0
9	50	94.0	112.2	121.5	131.5	181.0
10	74	70.0	90.5	103.0	119.0	213.0
11	26	76.0	92.5	98.0	108.8	149.0
12	77	86.0	105.0	113.0	123.0	180.0
14	30	78.0	91.2	98.5	107.8	146.0

Table S10. Mean ACC in first 30 min.

Subject ID	N	Min	Q1	Median	Q3	Max
1	36	62.9	63.4	63.8	64.1	65.2
2	43	63.6	64.7	65.1	65.4	65.8
4	14	63.9	64.8	65.0	65.1	65.9
5	42	62.9	63.3	63.6	64.1	66.0
8	26	63.0	63.1	63.3	63.5	65.3
9	48	64.7	65.7	65.9	66.1	66.4
10	74	62.3	64.4	64.8	65.1	65.8
11	38	62.1	62.9	63.3	64.2	66.1
12	74	63.5	64.9	65.3	65.6	67.8
14	28	62.9	64.2	64.7	65.2	67.7

Table S11. Comparison between random-intercept and random-slope mixed-effects models for peak glucose prediction.

	Random Intercept	Random Slope
AIC	2691.42	2665.33
BIC	2735.62	2716.90
Log-likelihood	-1333.71	-1318.66
Group variance	38.37	38.24
Net carbohydrate slope variance	–	113.71
Group $\times$ net carbohydrate covariance	–	26.91

The random-slope model estimated a significant positive fixed effect of within-person net carbohydrate intake ($\beta = 14.52$, $p < .001$), indicating that meals containing higher-than-usual net carbohydrate content were associated with higher peak glucose responses. Furthermore, the non-zero random-slope variance indicated that the magnitude of this association varied substantially across participants, which suggests individualized carbohydrate sensitivity profiles.

3 EXPLORATORY MACRONUTRIENT INTERACTION ANALYSES

Exploratory interaction analyses was performed to examine whether the association between carbohydrate intake and postprandial glycemic responses depended on the concurrent macronutrient composition of

Table S12. Comparison between random-intercept models and exploratory macronutrient interaction models. Interaction models included interaction terms between within-person net carbohydrate intake and dietary fiber, fat, and protein intake.

Outcome	Additive AIC	Interaction AIC	ΔAIC	Additive BIC	Interaction BIC	ΔBIC
Peak glucose	2691.42	2690.85	-0.57	2735.62	2746.10	10.48
Time to peak	2850.82	2854.92	4.10	2895.03	2910.17	15.15
AUC_{140}	4904.66	4906.66	2.00	4948.86	4961.91	13.05

Table S13. Estimated interaction effects between within-person net carbohydrate intake and other macronutrients in random-intercept mixed-effects models.

Outcome	Interaction Term	Coefficient (β)	p-value
Peak glucose	Net carbohydrate × dietary fiber	-1.17	0.427
	Net carbohydrate × fat	-1.56	0.493
	Net carbohydrate × protein	-1.62	0.496
Time to peak	Net carbohydrate × dietary fiber	-1.25	0.516
	Net carbohydrate × fat	1.92	0.526
	Net carbohydrate × protein	1.87	0.544
AUC_{140}	Net carbohydrate × dietary fiber	-103.75	0.102
	Net carbohydrate × fat	-35.59	0.718
	Net carbohydrate × protein	39.32	0.700

the same meal using random-intercept mixed-effects models. Specifically, interaction terms between net carbohydrate intake and dietary fiber, fat, and protein intake were added to the random-intercept models. Model fit was compared using AIC and BIC, where lower values indicate better fit after accounting for model complexity.

Overall, adding macronutrient interaction terms did not meaningfully improve model fit across the three primary glycemic outcomes (Table S12). For peak glucose, the interaction model showed only a negligible improvement in AIC (ΔAIC = -0.57), while BIC favored the simpler model (ΔBIC = 10.48). For time to peak and AUC_{140} mg/dL, both AIC and BIC favored the simpler models. Most interaction terms were non-significant. However, the interaction between net carbohydrate and dietary fiber showed consistently negative coefficients across outcomes, with the strongest trend observed for AUC_{140} ($\beta = -103.75$, $p = 0.102$). This pattern suggests that dietary fiber may attenuate carbohydrate-related glycemic excursions, although the results were not statistically significant. Given the limited sample size and reduced power for detecting interaction effects, these analyses should be interpreted as exploratory and hypothesis-generating.
